# Understanding the Unusual Response to High Pressure in KBe_2_BO_3_F_2_

**DOI:** 10.1038/s41598-017-04323-2

**Published:** 2017-06-22

**Authors:** D. H. Yu, M. Avdeev, D. H. Sun, L. Q. Huston, Thomas B. Shiell, Q. B. Sun, T. Lu, Q. Gu, H. Liu, J. E. Bradby, N. Yie, Y. Liu, J. Y. Wang, G. J. McIntyre

**Affiliations:** 1 Australian Centre for Neutron Scattering, Australian Nuclear Science and Technology Organisation, Lucas Heights, NSW 2234 Australia; 2 State Key Laboratory of Crystal Materials, Shandong University, Jinan, 250100 P.R. China; 3 Research School of Physics and Engineering, The Australian National University, Canberra, ACT 2601 Australia; 4 Research School of Chemistry, The Australian National University, Canberra, ACT 2601 Australia; 5 Australian Synchrotron, Clayton, VIC 3168 Australia; 6 Fujian Institute of Research on the Structure of Matter, Chinese Academy of Sciences, Fuzhou, 350002 China; 7 School of Chemistry, The University of Sydney, Sydney, NSW 2006 Australia

## Abstract

Strong anisotropic compression with pressure on the remarkable non-linear optical material KBe_2_BO_3_F_2_ has been observed with the linear compression coefficient along the *c* axis found to be about 40 times larger than that along the *a* axis. An unusual non-monotonic pressure response was observed for the *a* lattice parameter. The derived bulk modulus of 31 ± 1 GPa indicates that KBe_2_BO_3_F_2_ is a very soft oxide material yet with stable structure up to 45 GPa. A combination of high-pressure synchrotron powder X-ray diffraction, high-pressure Raman spectroscopy, and Density Functional Theory calculations points to the mechanism for the unusual pressure response being due to the competition between the K-F bond length and K-F-K bond angle and the coupling between the stretching and twisting vibration modes.

## Introduction

KBe_2_BO_3_F_2_ (KBBF) single crystal is a well-known deep ultraviolet (DUV) nonlinear optical material^1, 2^. Ultraviolet laser light with wavelength less than 180 nm has been generated through fourth, fifth, or sixth harmonic generation^1^ with single-crystalline KBBF, the only material with this capability identified to date. This has thus offered many important applications in fields such as medical laser surgery, precise photolithography, material processing, and super-high-resolution photoemission spectroscopy. For example, a deep-UV laser generated from single-crystalline KBBF made possible measurement of the energy gap and its dependence on electron propagation directions in unconventional superconductors, realized from correlated *d*- and *f*-electrons^1, 3, 4^. In addition to the unique non-linear optical properties, other new properties of KBBF crystal like negative thermal expansion (NTE), negative area compressibility (NAC), and the associated potential applications such as smart strain conversion and acoustical-optical coupling devices have been explored recently^5, 6^. These abnormal behaviors are believed to be attributed to the concerted structural distortion of the [BeO_3_F] tetrahedra in the two-dimensional [Be_2_BO_3_F_2_] framework under external stimulus of temperature and/or pressure^6^.

From the materials synthesis point of view, large KBBF crystals with thickness greater than 4 mm are very difficult to synthesize using the flux method (flux-KBBF) due to the layered crystal structure. This severely hinders the applications of this material. Although larger crystals (hydro-KBBF) could be fabricated through a hydrothermal process^7^ the second-harmonic-generation (SHG) efficiency of the resultant crystal is significantly lower than that of KBBF crystals (flux-KBBF) grown with the flux method^2^. Single-crystal X-ray diffraction (XRD) revealed that the hydro-KBBF crystal belongs to a different space group ($R\overline{3}c$) as compared with the *R*32 structure for the flux-KBBF^5^. It was further confirmed that a hydro-KBBF powder sample contains a mixture of the above two structures with about 20% of *R*32 and 80% of $R\overline{3}c$. This phase impurity leads to the lower SHG efficiency because the dominant $R\overline{3}c$ component has no SHG effect due to its centro-symmetric structure^5^. Thus it becomes necessary to study possible phase transitions between these two structures under external stimuli such as temperature and pressure. Early temperature-dependent XRD investigation did not observe any phase transition for either flux-KBBF or hydro-KBBF for temperature up to 1000 K. A strong anisotropic lattice thermal expansion was observed as a function of temperature, including the observation of NTE in the *ab* plane from room temperature up to 475K^5^.

In this paper, we report our investigation on the high-pressure response of KBBF using synchrotron powder X-ray diffraction (PXRD), Raman Spectroscopy (RS), and Density Functional Theory (DFT) calculations, aiming to provide a better understanding of the mechanism for the NAC property of this material.

## Results and Discussion

### High-pressure PXRD

**Figure 1:**
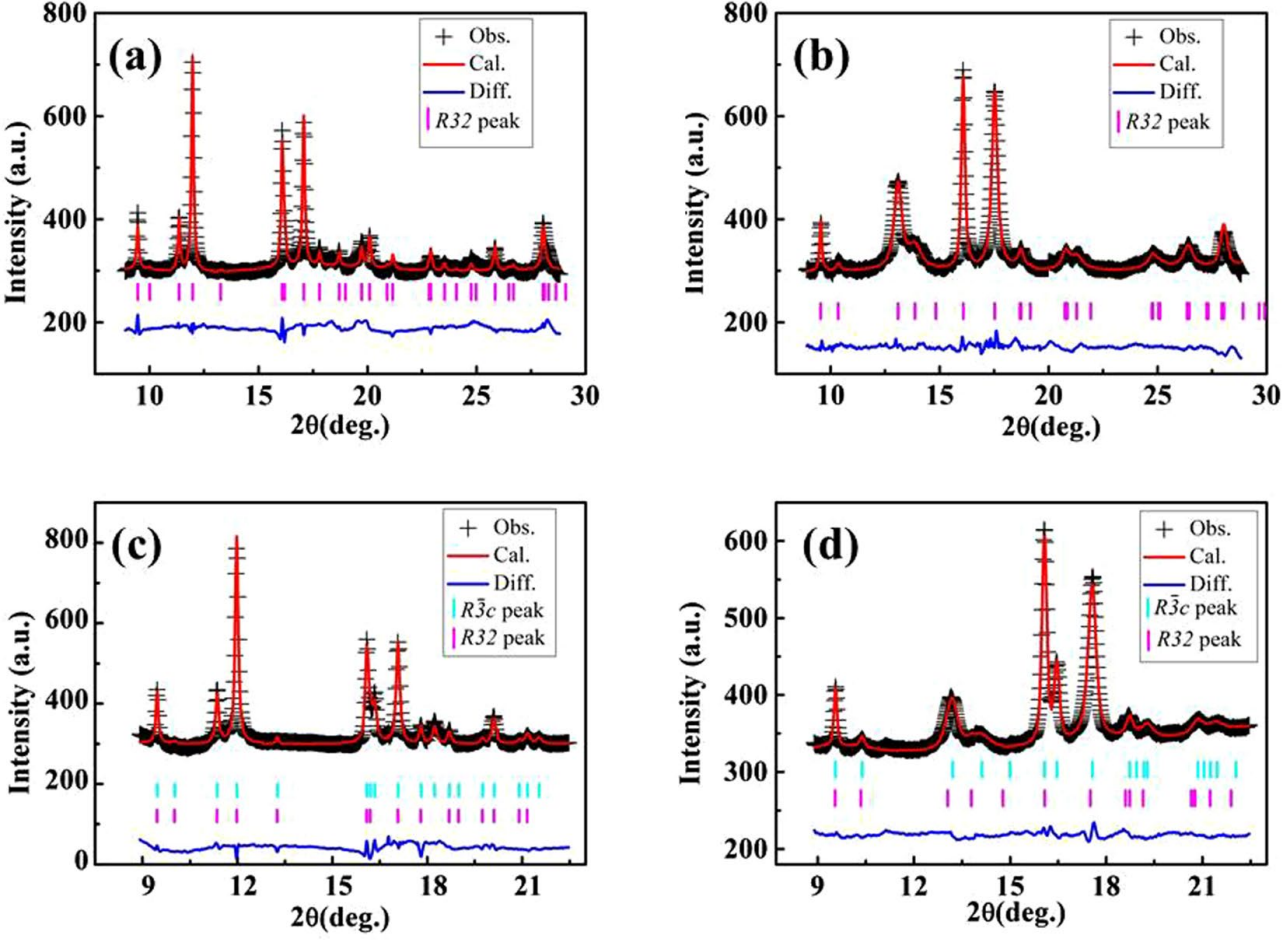
Synchrotron PXRD patterns for flux-KBBF at ambient pressure (**a**) and 9.7 GPa (**b**), and hydro-KBBF at ambient pressure (**c**) and 10.2 GPa (**d**). Plus (+) symbols: the observed patterns; Red lines: the refined patterns; Blue lines: the difference between observed and refined patterns; Magenta and cyan vertical ticks indicate the calculated position of Bragg reflections for *R32* and $R\overline{3}c$ structures, respectively. A sloping background has been removed from all the experimental diffraction patterns.

Synchrotron PXRD patterns were collected from ambient pressure (AP) to 9.7 GPa and 10.2 GPa for flux-KBBF and hydro-KBBF, respectively. Figure 1(a) and (b) show the experimental diffraction patterns together with the refinement results corresponding to AP and 9.7 GPa, respectively, for flux-KBBF. For hydro-KBBF, the refinement starts with a mixture of 20%*R*32 and 80%$R\overline{3}c$ structures based on the previous studies on the same sample^5^. The corresponding *R32* parameters obtained from the pressure-dependent flux-KBBF measurements are used for the *R32* component in the subsequent refinement of pressure-dependent XRD data for the hydro-KBBF sample. During the refinement, variation of the ratio between *R*32 and $R\overline{3}c$ does not improve the refinement significantly, and thus the ratio remains unchanged over the pressure range covered. The experimental diffraction patterns and the corresponding refinement results at AP and 10.2 GPa are displayed in Fig. 1(c) and (d), respectively. The relevant structure information for the two samples from the refinements can be found in Table 1.

**Table 1:** The refined structure parameters of KBBF.

	flux-KBBF		hydro-KBBF	
**Pressure/GPa**	AP	9.7	AP	10.2
**Space group**	*R32*	*R32*	$R\overline{3}c$	$R\overline{3}c$
***a*** **/(Å)**	4.429 (2)	4.436 (2)	4.429 (1)	4.440 (3)
***c*** **/(Å)**	18.60 (2)	15.43 (2)	37.11 (3)	30.28 (4)
***V*** **/(Å** ^**3**^ **)**	316.0 (3)	262.8 (3)	630.6 (5)	516.8 (9)
**R**_**B**_ **(%)**	6.14	6.21	7.05	7.13
**R**_**F**_ **(%)**	5.39	5.52	4.67	4.86

**Figure 2:**
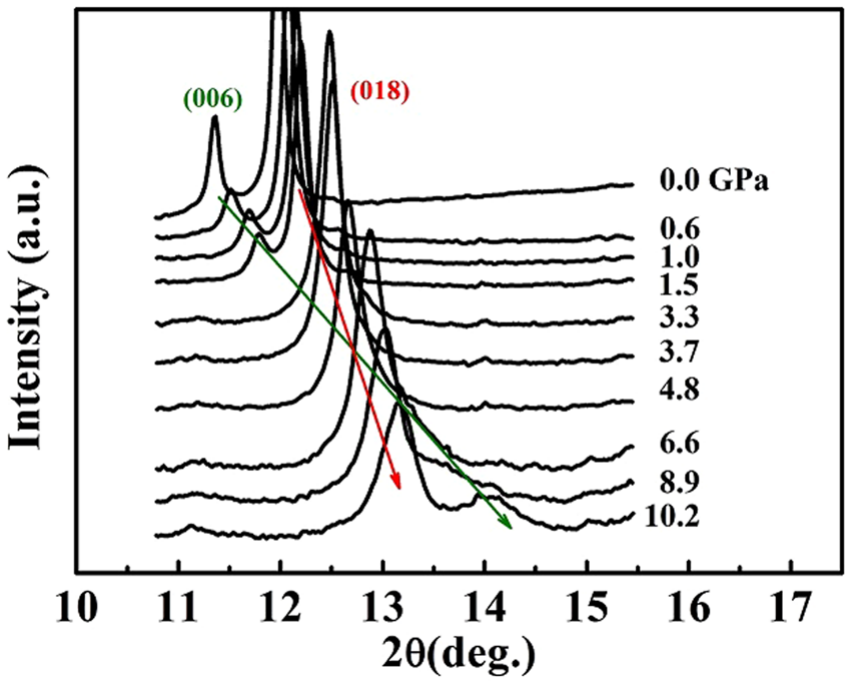
The pressure-dependent XRD patterns show the dramatic changes of the (006) reflection (green arrow) with pressure, in contrast with the (018) reflection (red arrow) in the *2θ* range from 10° to 16° for hydro-KBBF.

Significant changes in the diffraction patterns for both phases were observed, for example the position and intensity for the (006) reflection change dramatically with pressure in contrast to the (018) reflection, as shown in Fig. 2. The (006) peak is well separated from the (018) peak at low pressures. After overlapping with the (018) peak in the pressure range of 3 to 6 GPa, the (006) reflection shows up on the other side of the (018) reflection at high pressures. This indicates the strong anisotropic response to pressure. However there is no evidence showing any phase transition over the pressure range covered here, consistent with a previous report^6^.

**Figure 3:**
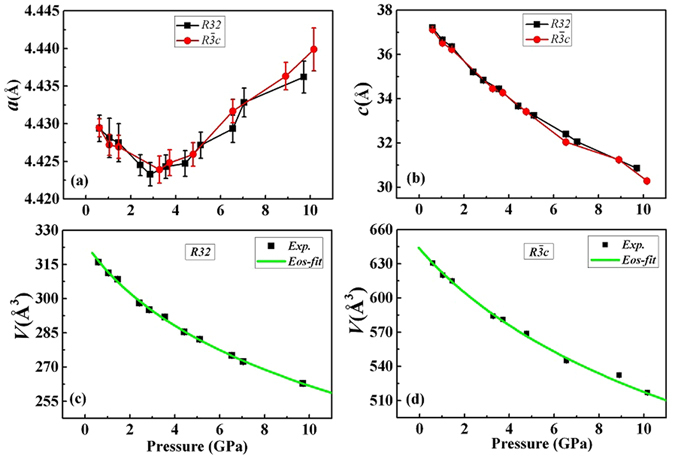
The pressure dependences of the lattice parameters *a*, *c*, and the volumes *V* of the *R32* and the $R\overline{3}c$ KBBF are shown in (**a**,**b**,**c** and **d**), respectively. The green lines in (**c**) and (**d**) represent the best fit of the second-order Birch-Murnaghan equation of state for *R32* and $R\overline{3}c$, respectively.

The pressure dependences of the lattice parameters *a, c*, and the unit-cell volume *V* of the two KBBF structures are plotted in Fig. 3. The *c* parameter of the *R32* structure is doubled for easy comparison with that of the $R\overline{3}c$ structure. Very similar pressure dependence is observed for both structures. While the *c* lattice parameter and volume *V* decrease with increasing pressure over the whole pressure range, the lattice parameter *a* decreases first until 3 GPa, then increases with increasing pressure. This non-monotonic pressure dependence was not noted in the earlier report for KBBF in this pressure range, however similar non-monotonic behavior was indeed observed for RbBe_2_BO_3_F_2_ and CsBe_2_BO_3_F_2_ with the same structure type as KBBF. We will come back to discuss this unusual pressure dependence later. The pressure range showing a NAC effect (in the *ab* plane) is from 3 GPa to 10 GPa, in contrast to the previous observation showing the effect from 0.22 GPa to 6.39 GPa^6^.

**Figure 4:**
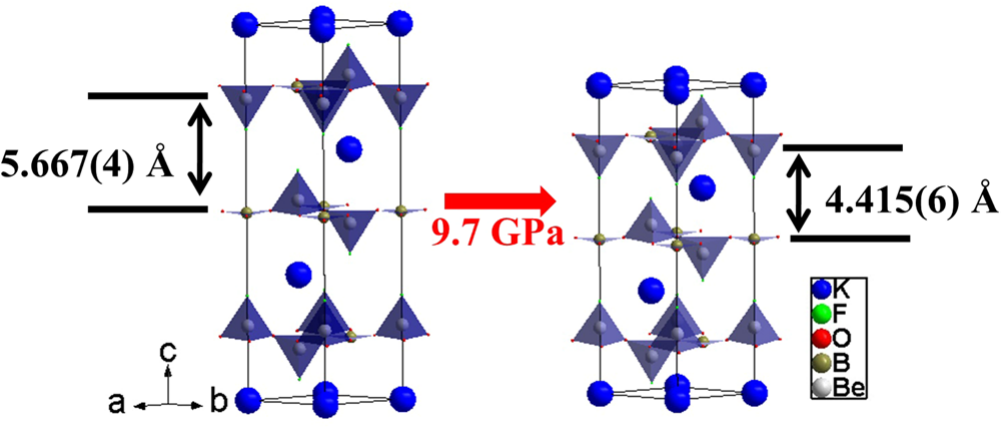
Structural schematics showing the large compression (more than 20%) along the *c* axis from AP to 9.7 GPa for flux-KBBF.

We can define the compression coefficient along different directions as:

1 $$\mathop{{\rm{\sigma }}}\limits^{{\rm{-}}}{\rm{(}}{{\rm{P}}}_{{\rm{0}}}{\rm{\to }}{\rm{P}}{\rm{)}}{\rm{=}}{\rm{-}}\frac{{\rm{\Delta }}{\rm{L}}}{{{\rm{L}}}_{{\rm{0}}}}\frac{{\rm{1}}}{{\rm{\Delta }}{\rm{P}}}$$

where $\mathop{{\rm{\sigma }}}\limits^{{\rm{-}}}{\rm{(}}{{\rm{P}}}_{{\rm{0}}}{\rm{\to }}{\rm{P}}{\rm{)}}$ is the compression coefficient over the pressure range from P_0_ to P, and the minus sign represents the compression, L_0_ is the lattice parameter at P_0_, and ΔL = L − L_0_ is the change of a lattice parameter corresponding to the pressure change of ΔP = P − P_0_. The value of ΔL/ΔP is obtained by a linear fitting of the lattice parameter as a function of pressure over the relevant pressure range. The results for both structures agree within error, thus we take the final results as the averaged values over the two structures. The derived compression coefficients along the *a* and *c* directions are σ_*a*_ = 5.2(8) × 10^−4^/GPa (AP to 3 GPa), σ_*a*_ = −5.0(6) × 10^−4^/GPa (3 to 10 GPa) and σ_*c*_ = 1.9(2) × 10^−2^/GPa (AP to 10 GPa). These values agree very well with what has been reported earlier^6^. Such large anisotropic compression, σ_*c*_ = 38 × σ_*a*_, is a direct reflection of the intrinsic anisotropic structural bonding property. The strong covalent bonding within the layers formed by the two-dimensional [Be_3_B_3_O_6_] network is responsible for low compressibility of the *ab* plane. On the other hand, the much weaker ionic inter-layer bonding through K atoms leads to the significant large compression coefficients observed along the *c* axis. As shown in Fig. 4 for flux-KBBF, the inter-layer distance is changed from 5.667(4) Å to 4.415(6) Å, with more than 20% decrease as the pressure is changed from AP to 9.7 GPa. However, the change of the *a* lattice parameter is less than 1% in this pressure range.

Though dramatically different compressibility is measured between intra-layer and inter-layer directions, the unit-cell volume decreases with increasing pressure for both structures as shown in Fig. 3(c) and (d). Through a least-squares fitting of the experimental volume-pressure dependence with a second-order Birch-Murnaghan equation of state (EOS)^8^ for both KBBF structures, as shown by the green lines in Fig. 3(c) and (d), we derived the following parameters: *V*_*0*_ = 320(1) Å^3^, *K*_*0*_ = 32(1) GPa for the *R32* structure and *V*_*0*_ = 641(3) Å^3^, *K*_*0*_ = 31(1) GPa for the $R\overline{3}c$ structure. These parameters correspond to the zero-pressure volume and bulk modulus, respectively. To the best of our knowledge, these values of bulk modulus are among the lowest values found for oxide materials. As a comparison, the bulk modulus for the (NH_4_)_2_V_3_O_8_ layer structure is 35.1(7) GPa^9^. For (NH_4_)_2_V_3_O_8_, the layers containing VO_x_ polyhedra are aligned to each other along the *c* axis, and high pressure pushes the neighboring layers closer enough to form VO_x+1_ polyhedra resulting in a new high-pressure phase. However for the KBBF structure, the (BO_3_)^3−^ triangles and (BeO_3_F)^5−^ polyhedra in the neighboring layers are not aligned along the *c* axis, thus there is much more space for them to move. This structural difference is the principal reason why KBBF is more compressible than (NH_4_)_2_V_3_O_8_.

**Figure 5:**
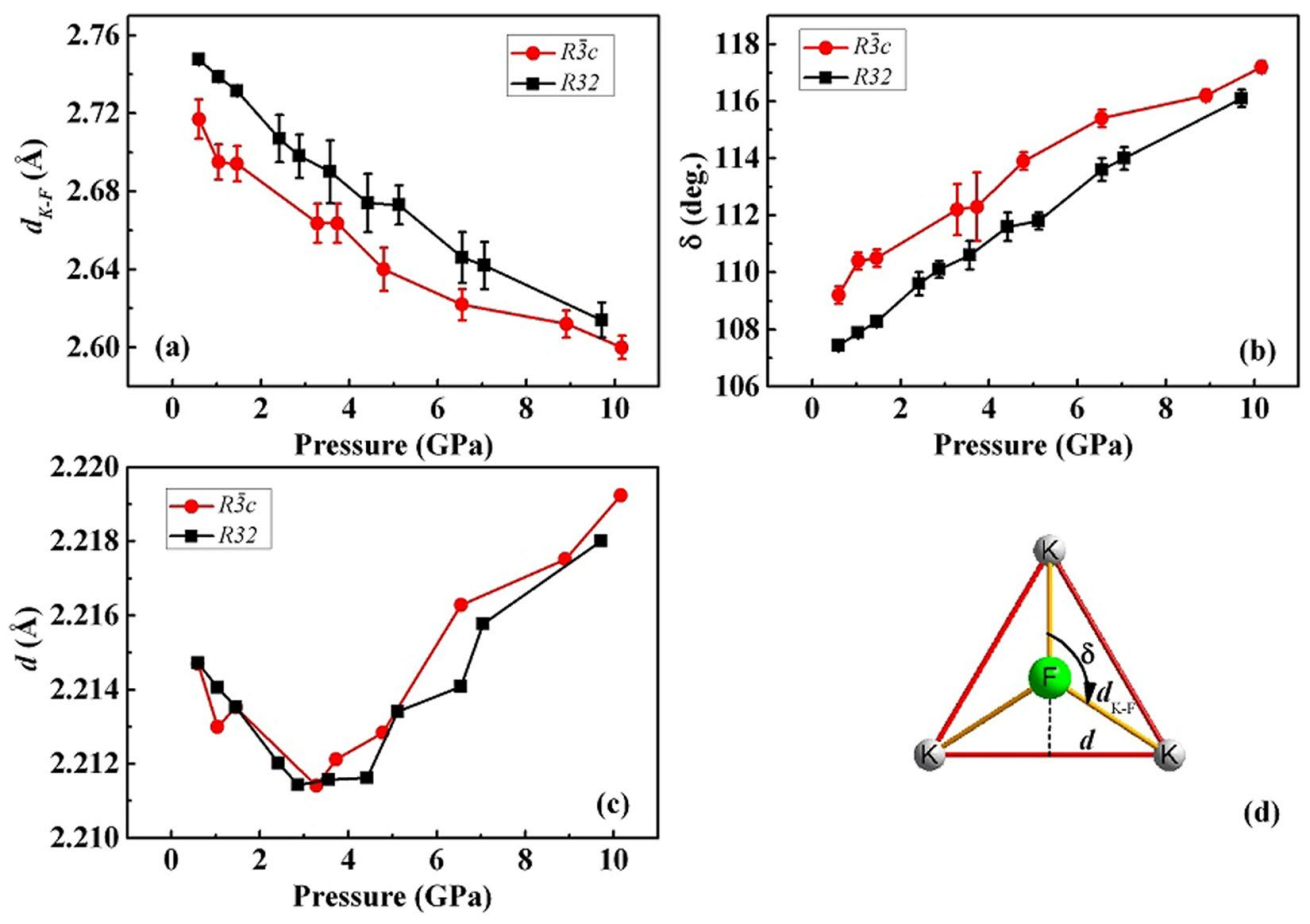
The pressure dependence of the K-F bond length *d*_*K-F*_, the angle δ of K-F-K, and the projection *d* of the K-F bond length along the *a* axis are shown in (**a**,**b** and **c**) respectively. The sketch (**d**) shows the relationship among these parameters of bond length, angle, and projection.

Now we address the unusual pressure dependence of the lattice parameter *a* as shown in Fig. 3(a). For this purpose, we evaluate the changes of the K-F bond length (*d*_*K-F*_), the K-F-K angle (δ), and the projection of the *d*_*K-F*_ on the *a* axis as a function of pressure. The projection is given by *d* = *d*_*K-F*_ * *sin(δ/2)* as indicated in Fig. 5(d). The results are presented in Fig. 5. It is clearly seen that the distance *d*_*K-F*_ decreases with increasing pressure while the angle δ shows an opposite trend. Changes of about −5% and 7% in *d*_*K-F*_ and δ, respectively, together cause −0.2% and 0.3% changes in the projection *d* for the two pressure ranges below and above 3 GPa. These changes are the same as that of the lattice parameter *a* in these pressure ranges. The competition between the distance and the angle gives rise to the non-monotonic pressure dependence of the lattice parameter *a*. Initial compression is mainly due to the decreasing interatomic distances with pressure. After 3 GPa pressure, the contribution from the angle δ becomes dominant, thus causing the lattice parameter *a* to increase with pressure. It is interesting to note that the Be-O bond length and O-Be-O bond angle have very little pressure dependence, thus contributing considerably less to the pressure variation of the *a* lattice parameter. While it was reported previously that the competition between these two parameters involving Be and O caused the non-monotonic temperature dependence of the *a* lattice parameter^5^, it is clear that it is the competition between the K-F bond length and K-F-K bond angle that contributes directly to the concerted distortion of the [BeO_3_F] tetrahedra within the [Be_2_BO_3_F_2_] layer under pressure, consequently causing the unique NAC in this material.

**Figure 6:**
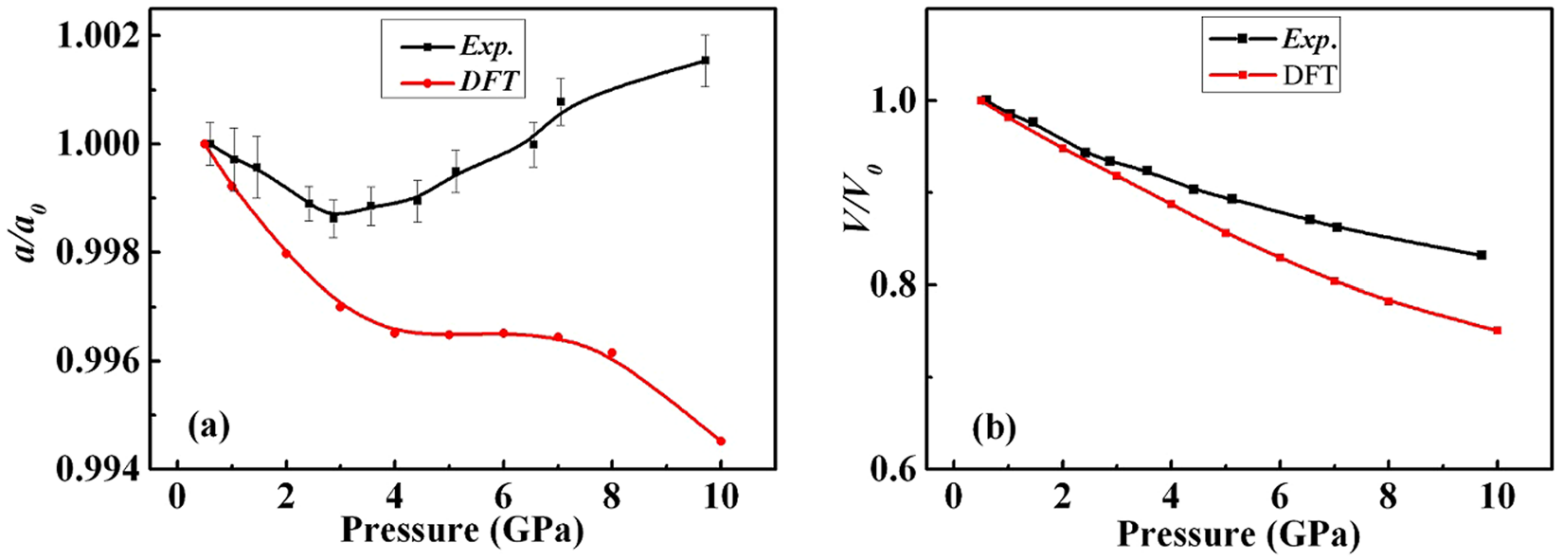
The relative changes of lattice parameter *a/a*_*0*_ (**a**) and volume *V/V*_*0*_ (**b**), where *a*_*0*_ and *V*_*0*_ correspond to the values at 0.5 GPa pressure for flux-KBBF. Black squares are experimental results and red dots are the DFT calculations. Solid lines serve as guides to the eye only. Note that the experimental error bars in (**b**) are smaller than the symbols.

Figure 6 shows the comparison between the experimental and DFT-calculated lattice parameter *a* and unit-cell volume *V* relative to the initial values corresponding to 0.5 GPa pressure for flux-KBBF. Though the exact pressure dependence of lattice parameter *a* is not well reproduced from the DFT calculation, the non-monotonic behavior is predicted by the calculation qualitatively. We note that some discrepancy between the calculations and experiment is inevitable as in the former a truly isotropic stress is modelled while in the latter that condition is maintained only at rather low pressures. Other microstructural effects, e.g. intergrain stresses, may possibly develop in the sample as pressure increases. Especially in this case KBBF has a strong layer structure and no matter how fine the powder sample is, it always contains small flakes. These factors may cause deviation from the true hydrostatic condition in the measurements. Taking these effects into account, a reasonable agreement between theory and experiment is demonstrated for the unit-cell volume as a function of pressure. The bulk modulus *K*_*0*_ from the DFT is 23 GPa which is lower than the experimental result. The corresponding DFT results up to 45 GPa have been included in Fig. S1 in supporting information.

### High-pressure Raman spectroscopy

**Figure 7:**
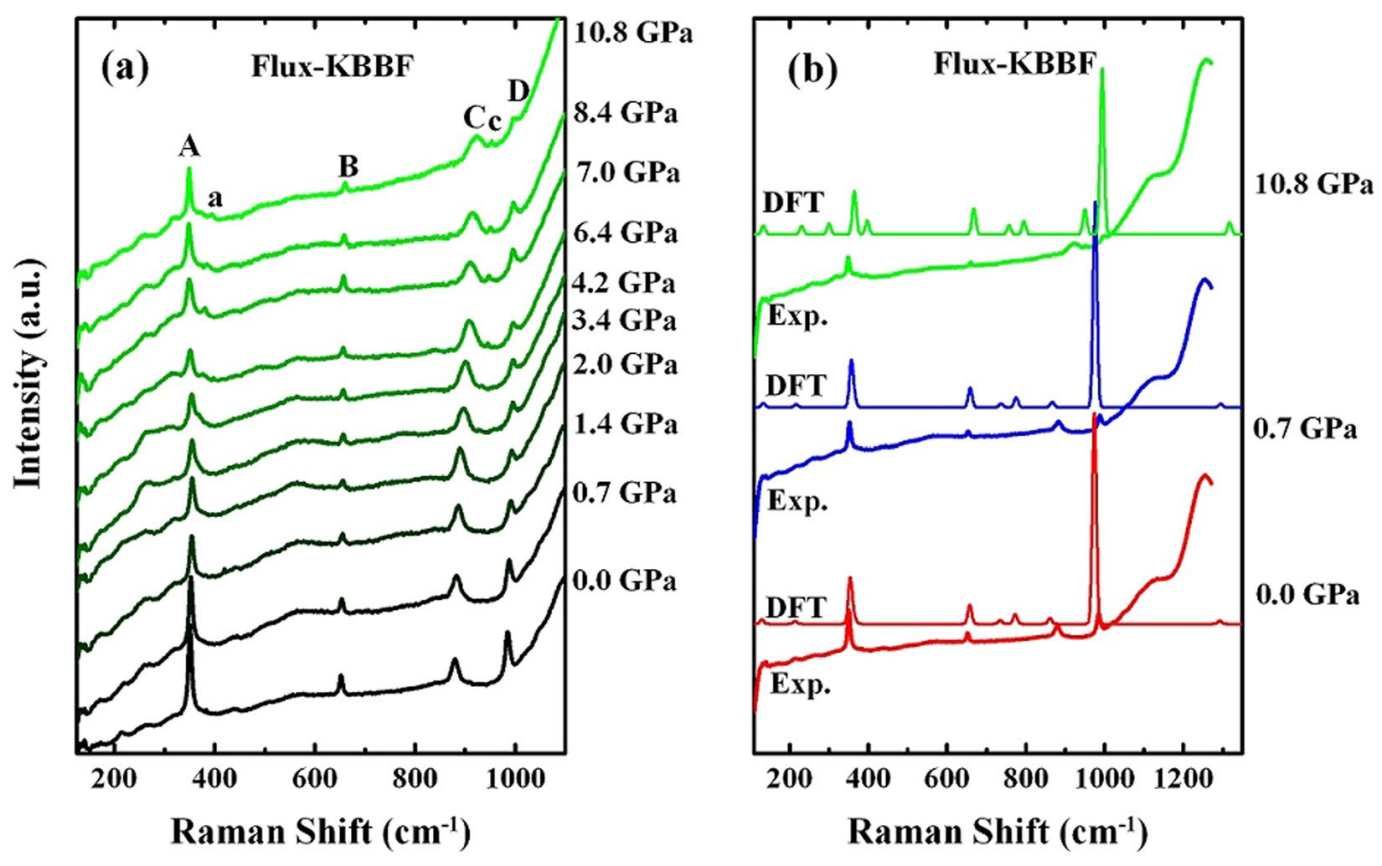
Raman spectra as a function of pressure are shown in (**a**) and the comparisons between DFT and experiment results are indicated in (**b**) for flux-KBBF.

For a better understanding of the structural response to high pressure, we carried out Raman spectroscopy studies as a function of pressure up to 45 GPa, for both flux-KBBF and hydro-KBBF samples. Very similar spectra have been observed for both samples, as shown in Fig. 7 for flux-KBBF for pressure up to 10 GPa, while the corresponding results for hydro-KBBF are presented in Fig. S2 in supporting information. As seen in Fig. 7(a), six modes have been clearly observed in the range of 100 cm^−1^ to 1000 cm^−1^, marked by ‘A’, ‘a’, ‘B’, ‘C’, ‘c’ and ‘D’ in the figures. The peak ‘C’ is from the pressure transmission medium of methanol/ethanol, which overlaps with the KBBF peak ‘c’ at low pressures. The comparison between the experimental data and the DFT calculations for flux-KBBF is displayed in Fig. 7(b). The calculations reproduce the observed modes reasonably well in terms of peak positions. Several extra modes are predicted but not observed, most likely due to the low intensity of these modes.

In a first inspection of the spectra evolution with pressure, it is noticed that mode a and mode c start showing up at pressures above 3 GPa, indicating a possible phase transition. However, the DFT calculations predict these modes even for the ambient-pressure crystal structure. This indicates that the appearance of mode a from mode A is possibly due to different pressure dispersion of these two modes. The mode c separates from the methanol/ethanol peak ‘C’ due to strong pressure dispersion at high pressures.

**Figure 8:**
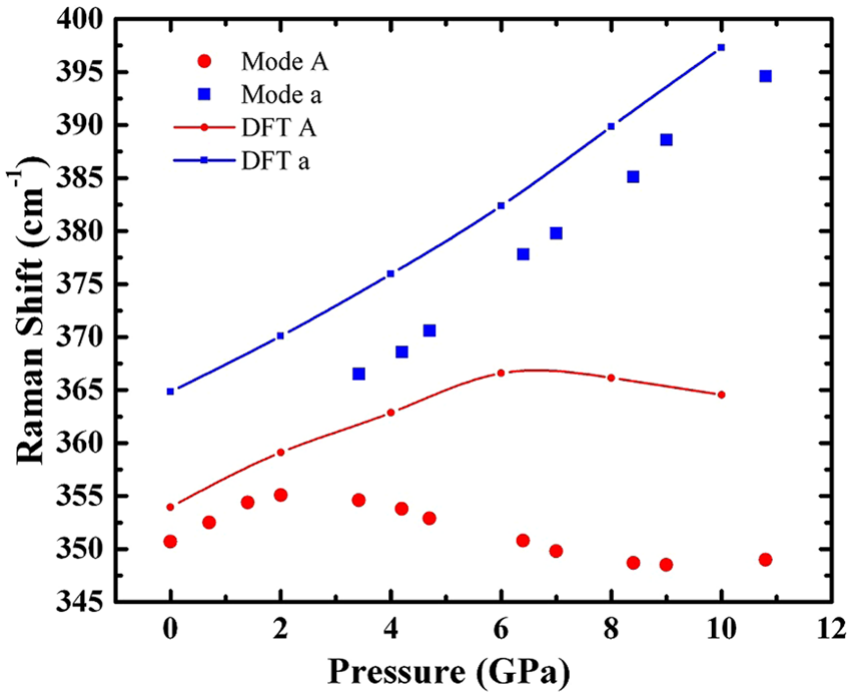
The frequencies of Raman mode A and mode a versus pressure for flux-KBBF. Red solid circles and blue solid squares represent experimental values, while the corresponding lines are the results from DFT calculations.

The frequencies of the modes A and a versus pressure (up to 10 GPa) are shown in Fig. 8 for flux-KBBF. The DFT calculation results are also included for comparison. The pressure dependences for the observed five modes from both samples are presented in Fig. S3 in supporting information for the pressure range up to 10 GPa. The corresponding results for pressure up to 45 GPa are presented in Fig. S4, while the experimental Raman spectra versus pressure are presented in Fig. S5 in supporting information. As shown in Fig. S5, there is no extra mode observed at 45.18 GPa pressure and this indicates that no phase transition occurs for pressure up to 45 GPa. The experimental results are very similar for both samples, although quite different trends with pressure are observed for each individual mode. As shown in Fig. 8, mode A first hardens (increases in frequency) with increasing pressure up to around 3 GPa, then softens all the way up to 8 GPa followed by a kind of plateau until 10 GPa, then almost a linear increase with pressure up to 45 GPa (Fig. S4). The DFT calculation predicts a similar behavior but with the hard-soft switching point shifting to high pressure (around 6 GPa) and the soft-hard switching point to 15 GPa (Fig. S4). The frequency of mode a shows a monotonic increasing trend with pressure from 3 GPa. The other modes all show increasing frequency versus pressure with a slight variation at low pressure which is associated with the mode A. DFT calculations also confirm these observations qualitatively.

**Figure 9:**
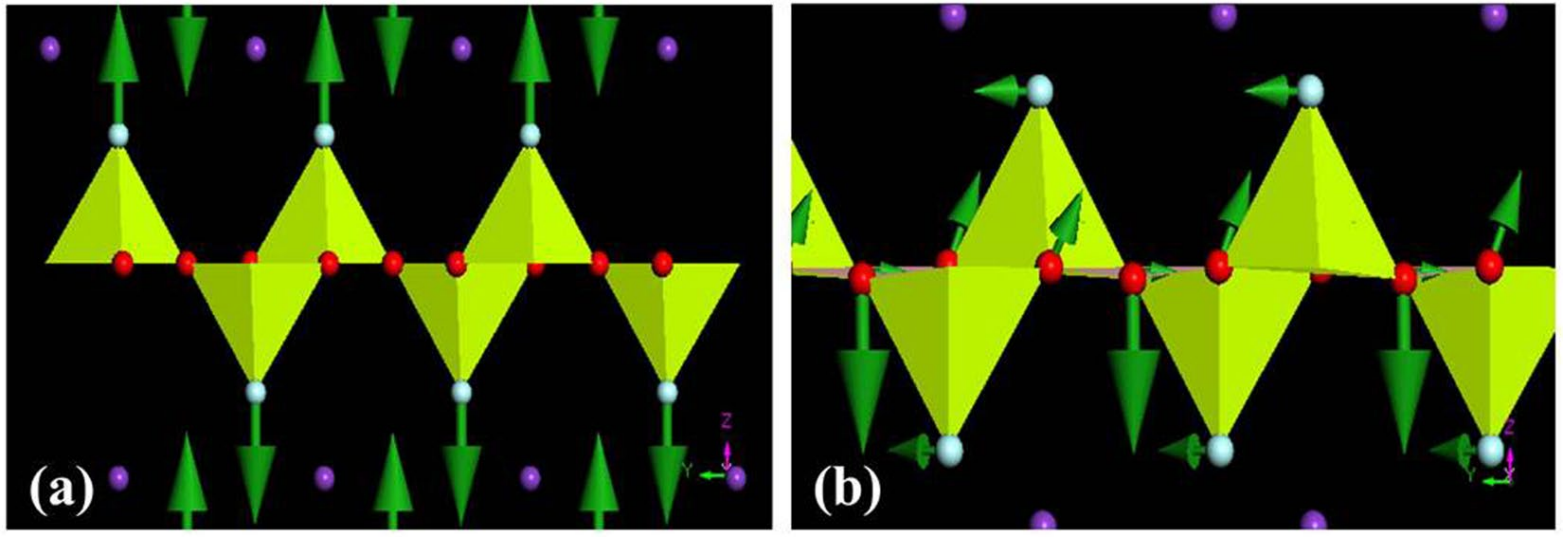
The stretching mode A (**a**) and twisting mode a (**b**) are shown for flux-KBBF at 4 GPa pressure.

Mode A corresponds to the stretching of [BeO_3_F] tetrahedra along the crystallographic *c* axis, as shown in Fig. 9(a). The unusual pressure response of this mode directly correlates with the change of the *a* lattice parameter as a function of pressure as seen in Fig. 3(a). For pressure up to about 3 GPa, this stretching mode responds to pressure normally due to the easy compressibility along the *c* axis and associates with the normal pressure dependence of the *a* lattice parameter. With further increasing pressure above 3 GPa, the stretching mode becomes softer as a result of energy shifting to the twist mode a which has both in-plane and out-of-plane motions with respect to the plane of [Be_2_BO_3_F_2_], as shown in Fig. 9(b). The frequency increase of mode a correlates with the increase of the bond angle of K-F-K and thus contributes to the increase of the *a* lattice parameter with pressure. The unique pressure dependence of these two modes correlates strongly with the structure distortion under pressure. From a dynamic point of view, the coupling of these two vibrational modes causes the NAC effect in this material.

In Summary, the structural and dynamic responses to pressure of the non-linear optical material KBBF have been systematically investigated with the combination of synchrotron powder X-ray diffraction, Raman spectroscopy, and DFT calculations. A good understanding of the unusual pressure response has been achieved. Structurally, the interaction between the K-F bond length and K-F-K angle gives rise to the unusual non-monotonic pressure dependence of the *a* lattice parameter and the associated NAC effect in the *ab* plane. Dynamically, the coupling of the stretching mode and the twist mode of [BeO_3_F] tetrahedra is responsible for the unique pressure dependence. The same mechanism should apply to the similar phenomena observed in the isostructural materials RbBe_2_BO_3_F_2_ and CsBe_2_BO_3_F_2_^5^. This research demonstrates that KBBF is structurally stable against pressure up to 45 GPa even though it is a very soft oxide material.

## Methods

### Sample preparation

The flux- and hydro-KBBF powder samples were obtained from grinding numerous small single crystals. Flux-KBBF single crystals were prepared by a localized spontaneous nucleation process as described in detail elsewhere^10^. A mixture of KF-B_2_O_3_ was used as the flux with molar ratio of (1.0–1.5):(5.0):(0.7–1.2) (KBBF:KF:B_2_O_3_). The growth temperatures varied from 730 °C to 800 °C at different stages in the process. Hydro-KBBF single crystals were grown in aqueous solutions of KF and H_3_BO_3_ with seeds obtained through spontaneous nucleation^6^. The growth temperature was 360 °C and the filling rate of the autoclave was about 80%, resulting in a pressure of about 120 MPa.

### High-pressure PXRD

Synchrotron powder X-ray diffraction (PXRD) on KBBF powders under high pressure at room temperature was carried out at the Australian Synchrotron Powder Diffraction Beamline. The X-ray wavelength used was 0.6205 Å with a beam size of 140 μm × 140 μm. Pressure was generated by an Almax easyLab diamond-anvil cell and determined using the shift of the fluorescence line of the ruby^11^. A mixture of 4:1 methanol\ethanol was used as a pressure medium. The diffraction patterns were collected using a MarCCD 165 area detector. The intensity versus 2θ (from 6° to 21°) diffraction pattern was obtained by integrating the recorded 2D powder pattern using the program FIT2D^12^. Rietveld refinements were carried out on the obtained diffraction pattern with Fullprof^13^. A Thompson-Cox-Hastings pseudo-Voigt profile function was used. Strain model for the Laue class $\bar{3}m1$ was employed to account for the anisotropic strain broadening effect^14^.

### High-pressure Raman spectroscopy

Raman spectra were collected *in situ* using a Renishaw inVia reflex spectrometer system with a 1200 l/mm grating and a 785 nm laser with a spot size of approximately 20 μm^2^ and power of 240 mW. Each sample was loaded into a diamond anvil cell with a 400 μm culet size with a stainless steel gasket having a 200 μm diameter hole with a depth of 45 μm. A mixture of 4:1 methanol\ethanol was used as a pressure medium and pressure was determined by the shift in fluorescence of a small ruby ball placed into the cell^11^. Two measurements were performed with and without a pressure medium in the pressure cell for pressures up to 10 GPa and 45 GPa, respectively.

### Theoretical calculations

To gain insights into the mechanism of KBBF crystal structure evolution under pressure, we performed Density Functional Theory (DFT) *ab initio* calculations as implemented in the CASTEP code (version 6.0)^15^. Changes of the KBBF crystal structure and Raman spectra as a function of pressure were calculated using the Perdew–Burke–Ernzerhof (PBE) form of the Generalized Gradient Approximation (GGA) exchange-correlation functional^16^. The norm-conserving pseudopotentials were used to model electron-ion interactions both for structure relaxation and Raman spectra calculations. The Brillouin zone was sampled using the Monkhorst–Pack grid^17^ whose density was tested with respect to total energy. The 5 × 5 × 2 k-mesh (for the primitive-unit-cell setting) and plane-wave cut-off energy of 940 eV were found to produce a well-converged structure and were used for the final calculations. The total-energy convergence, maximum ionic force and displacement, and stress component tolerances were set at 5 × 10^−6^ eV/atom, 1 × 10^−2^ eV/atom, 5 × 10^−4^ Å, and 2 × 10^−2^ GPa, respectively.

## Electronic supplementary material

Supporting information
