# Supplementary material for: Understanding the Unusual Response to High Pressure in KBe2BO3F2
Source: Sci Rep. 2017 Jun 22;7:4027. doi: 10.1038/s41598-017-04323-2 (PMC5481392; doi:10.1038/s41598-017-04323-2)
Supplement: Supplementary file 1 — Supporting information [file 41598_2017_4323_MOESM1_ESM.pdf]

Understanding the Unusual Response to High Pressure in $KBe_2BO_3F_2$

D. H. Yu[1*], M. Avdeev[1,7], D. H. Sun[1,2], L. Q. Huston[3], Thomas B Shiell[3], Q. B. Sun[4], T. Lu[4], Q. Gu[5], H. Liu[2], J. E. Bradby[3], N. Yie[6], Y. Liu[4], J. Y. Wang[2] and G. J. McIntyre[1]

[1]*Australian Centre for Neutron Scattering, Australian Nuclear Science and Technology Organisation, Lucas Heights, NSW 2234, Australia*

[2]*State Key Laboratory of Crystal Materials, Shandong University, Jinan 250100, P.R. China*

[3]*Research School of Physics and Engineering, The Australian National University, Canberra, ACT 2601, Australia*

[4]*Research School of Chemistry, The Australian National University, Canberra, ACT 2601, Australia*

[5]*Australian Synchrotron, Clayton, VIC 3168, Australia*

[6] *Fujian Institute of Research on the Structure of Matter, Chinese Academy of Sciences, Fuzhou 350002, China*

*Corresponding authors: dyu@ansto.gov.au

Supporting Information

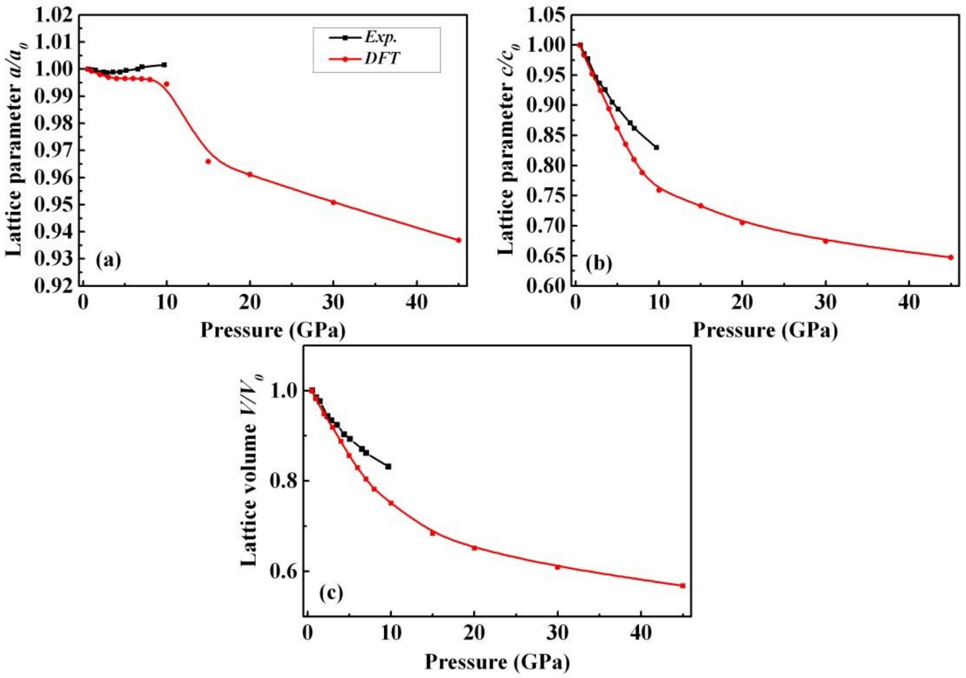

Fig. S1 Lattice parameters and unit-cell volume relative to the initial values as a function of pressure up to 45 GPa.

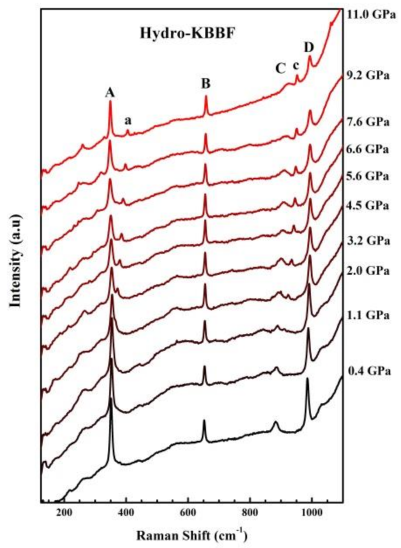

Fig. S2 Raman spectra versus pressure up to 10 GPa for hydro-KBBF.

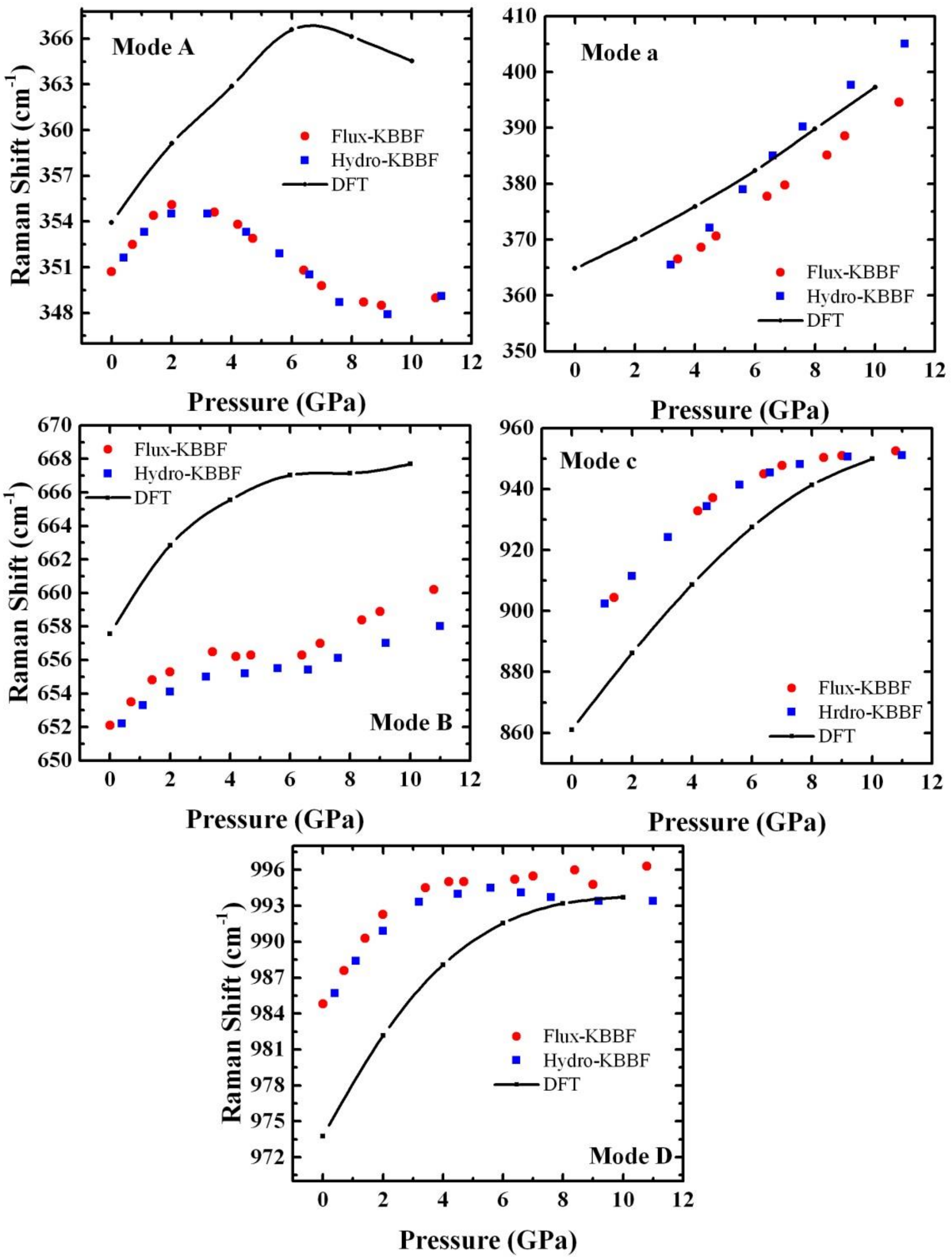

Fig. S3 The pressure dependences of the Raman frequencies for the five observed modes are presented for flux-KBBF and hydro-KBBF. The corresponding DFT results are also shown for comparison.

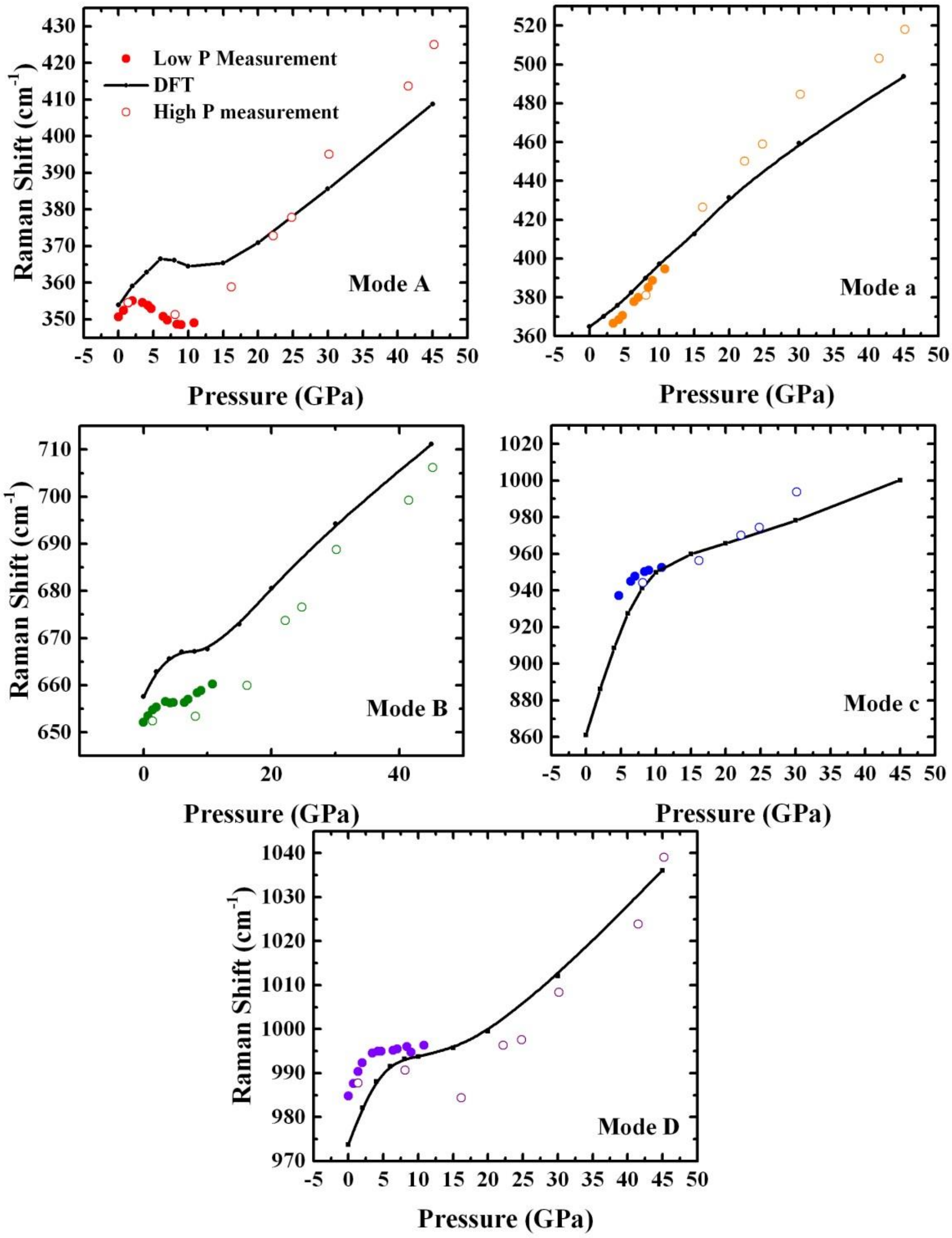

Fig. S4 The pressure dependences of the Raman frequencies for the five observed modes are presented for pressure up to 45 GPa for flux-KBBF. The corresponding DFT results are shown for comparison. Please note that the measurements up to 45 GPa were not performed under hydrostatic condition and an error bar of about 3 GPa in pressure is expected.

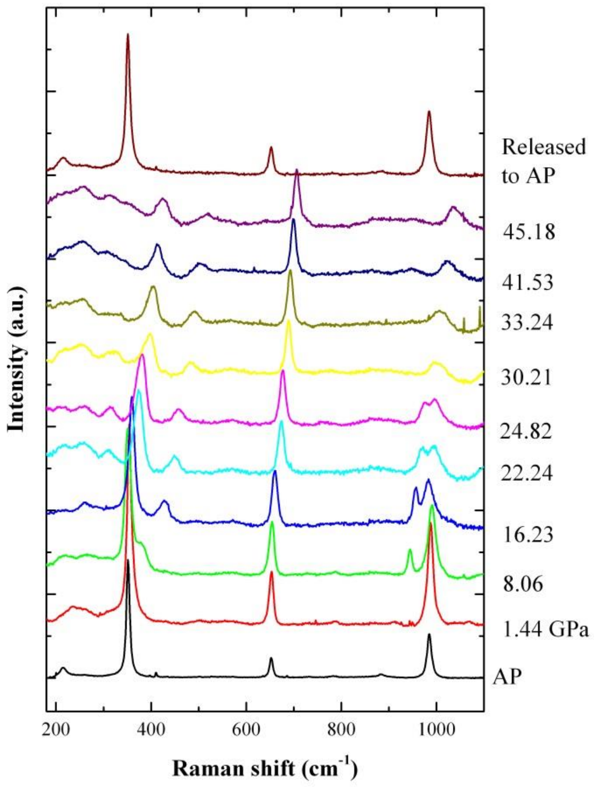

Fig. S5 The evolution of the experimental Raman spectra as a function of pressure is shown for pressure from ambient (AP) up to 45.18 GPa for flux-KBBF. Please note that the spectrum at AP after release from 45.18 GPa (top) is almost identical to the initial spectrum at AP (bottom). There is no extra peak observed at 45.18 GPa pressure.
